# Hidden MHC genetic diversity in the Iberian ibex (*Capra pyrenaica*)

**DOI:** 10.1186/s12863-018-0616-9

**Published:** 2018-05-08

**Authors:** Samer Angelone, Michael J. Jowers, Anna Rita Molinar Min, Paulino Fandos, Paloma Prieto, Mario Pasquetti, Francisco Javier Cano-Manuel, Gregorio Mentaberre, Jorge Ramón López Olvera, Arián Ráez-Bravo, José Espinosa, Jesús M. Pérez, Ramón C. Soriguer, Luca Rossi, José Enrique Granados

**Affiliations:** 1 Estación Biológica de Doñana, Consejo Superior de Investigaciones Científicas (CSIC), Avda. Américo Vespucio s/n, 41092 Sevilla, Spain; 2 Institute of Evolutionary Biology and Environmental Studies (IEU), University of Zurich, Winterthurerstrasse 190, Zurich, Switzerland; 3 CIBIO/ InBIO (Centro de Investigação em Biodiversidade e Recursos Genéticos), Universidade do Porto, Campus Agrario De Vairão, 4485-661 Vairão, Portugal; 4 Dipartimento di Scienze Veterinarie, Universita` degli Studi di Torino, Grugliasco, Italy; 5 Agencia de Medio Ambiente y Agua, E-41092 Sevilla, Isla de la Cartuja Spain; 6 Parque Natural Sierras de Cazorla, Segura y Las Villas, Martínez Falero11, E-23470 Cazorla, Jaén Spain; 7 Espacio Natural Sierra Nevada, Carretera Antigua de Sierra Nevada, Km 7, E-18071 Pinos Genil, Granada Spain; 8 Servei d’Ecopatologia de Fauna Salvatge (SEFAS), Departament de Medicina i Cirurgia Animals, Universitat Autònoma de Barcelona (UAB), E-08193 Bellaterra, Barcelona, Spain; 9 Departamento de Biología Animal, Biología Vegetal y Ecología, Universidad de Jaén, Campus Las Lagunillas, s.n., E-23071 Jaén, Spain

**Keywords:** *Capra pyrenaica hispanica*, *Capra pyrenaica victoriae*, *Capra aegagrus hircus*, Major histocompatibility complex (MHC), MHC DRB1, OLADRB1, Linkage disequilibrium, DRB-STR method, Sierras de Cazorla, Segura and las Villas Natural Park, Spain

## Abstract

**Background:**

Defining hidden genetic diversity within species is of great significance when attempting to maintain the evolutionary potential of natural populations and conduct appropriate management. Our hypothesis is that isolated (and eventually small) wild animal populations hide unexpected genetic diversity due to their maintenance of ancient polymorphisms or introgressions.

**Results:**

We tested this hypothesis using the Iberian ibex (*Capra pyrenaica*) as an example. Previous studies based on large sample sizes taken from its principal populations have revealed that the Iberian ibex has a remarkably small MHC DRB1 diversity (only six remnant alleles) as a result of recent population bottlenecks and a marked demographic decline that has led to the extinction of two recognized subspecies. Extending on the geographic range to include non-studied isolated Iberian ibex populations, we sequenced a new MHC DRB1 in what seemed three small isolated populations in Southern Spain (*n* = 132). The findings indicate a higher genetic diversity than previously reported in this important gene. The newly discovered allele, MHC DRB1*7, is identical to one reported in the domestic goat *C. aegagrus hircus*. Whether or not this is the result of ancient polymorphisms maintained by balancing selection or, alternatively, introgressions from domestic goats through hybridization needs to be clarified in future studies. However, hybridization between Iberian ibex and domestic goats has been reported in Spain and the fact that the newly discovered allele is only present in one of the small isolated populations and not in the others suggests introgression. The new discovered allele is not expected to increase fitness in *C. pyrenaica* since it generates the same protein as the existing MHC DRB1*6. Analysis of a microsatellite locus (OLADRB1) near the new MHC DRB1*7 gene reveals a linkage disequilibrium between these two loci. The allele OLADRB1, 187 bp in length, was unambiguously linked to the MHC DRB1*7 allele. This enabled us to perform a DRB-STR matching method for the recently discovered MHC allele.

**Conclusions:**

This finding is critical for the conservation of the Iberian ibex since it directly affects the identification of the units of this species that should be managed and conserved separately (Evolutionarily Significant Units).

**Electronic supplementary material:**

The online version of this article (10.1186/s12863-018-0616-9) contains supplementary material, which is available to authorized users.

## Background

Hidden genetic diversity, that is, unreported allelic diversity in already studied species or populations, is of great significance in the maintaining of the evolutionary potential of natural populations and the execution of appropriate management methods [1]. Cryptic genetic diversity is critical in conservation biology since it directly affects the identification of the units of species that need to be managed and conserved separately (Evolutionarily Significant Units, ESU) [2].

The major histocompatibility complex (MHC) plays a key part in the recognition of foreign antigen and the immune response to pathogens and parasites in vertebrates [3]. For this reason, MHC and immune gene variation are regarded as a barometer for the genetic health of wild populations [4]. High levels of allelic diversity have been found in MHC genes [5], which makes these closely linked genes some of the most polymorphic regions in the whole vertebrate genome [6]. Host-parasite co-evolution is assumed to maintain this level of polymorphism in the MHC loci [7], even though the molecular mechanisms involved in maintaining such extraordinary MHC polymorphism in vertebrates are still debated by epidemiologists, immunogeneticists and evolutionary biologists alike [8]. Nevertheless, many endangered and currently non-endangered species such as Arabian oryx (*Oryx leucoryx*), muskox (*Ovibos moschatus*), moose (*Alces alces*), fallow deer (*Dama dama*), beaver (*Castor fiber*), Asiatic lion (*Panthera leo persica*), cotton-top tamarin (*Saguinus oedipus*), cheetah (*Acinonyx jubatus*) and Tasmanian devil (*Sarcophilus harrisii*) all exhibit reduced allelic variation or even monomorphism at the MHC loci caused mainly by severe population bottlenecks [9–16].

Additionally, the well-known limited MHC variability in wild goats (genus *Capra*) may be related to its northerly distribution since allelic diversity at MHC DRB class II in wild ungulates decreases with increasing latitude, possibly either as a result of lower parasite diversity at high latitudes [9], proximity to the limit of the species’ range, and/or bottleneck effects provoked by recent declines in population size [17]. The low MHC variability in wild goats (genus *Capra*) potentially exposes their populations to collapse due either, among other stochastic events, to the introduction of pathogens or northward distribution shifts of pathogens triggered by climate warming [18].

Four subspecies of Iberian ibex are officially recognized [19, 20], of which two (*C. p. pyrenaica* and *C. p. lusitanica*) have recently become extinct. The surviving subspecies (*C. p. hispanica* and *C. p. victoriae*) have an allopatric distribution in the Iberian Peninsula [21]. Previous studies centred on the few main Iberian ibex populations have revealed that this ibex has remarkably low genetic variation at the class II MHC DRB1 gene, with only six different DRB1 alleles [22–24]. One of the alleles (MHC DRB1*4) became extinct with the extinction of the subspecies *C. p. pyrenaica*. The limited allelic variability of the DRB1 gene in the Iberian ibex is likely to be the direct result of its recent history of population bottlenecks and severe demographic decline [25, 26].

Our hypothesis is that small and isolated wild animal populations hide unexpected genetic diversity due to the maintenance of ancient polymorphisms or introgressions. Small and isolated population are much more exposed to introgression scenarios as a result of hybridization with domestic animals [26]. The aim of the present study was to test this hypothesis using the Iberian ibex as an example. We extended the sampling size to include small isolated populations ignored by previous studies. If our hypothesis (new MHC DRB1 alleles) is true, we will need to develop a simple and relatively inexpensive protocol for genotyping the newly discovered alleles. The method described by Alasaad et al. [23] is based on linkage disequilibrium analysis of a microsatellites locus (OLADRB1) and the MHC DRB1 gene. The OLADRB1 is located close to the MHC DRB1 gene [27] and hence the allele length polymorphism at OLADRB1 is usually unambiguously linked to a particular DRB1 allele; thus, sequencing the MHC DRB1 gene is not necessary.

## Methods

### Sample collection and DNA extraction

**Fig. 1:**
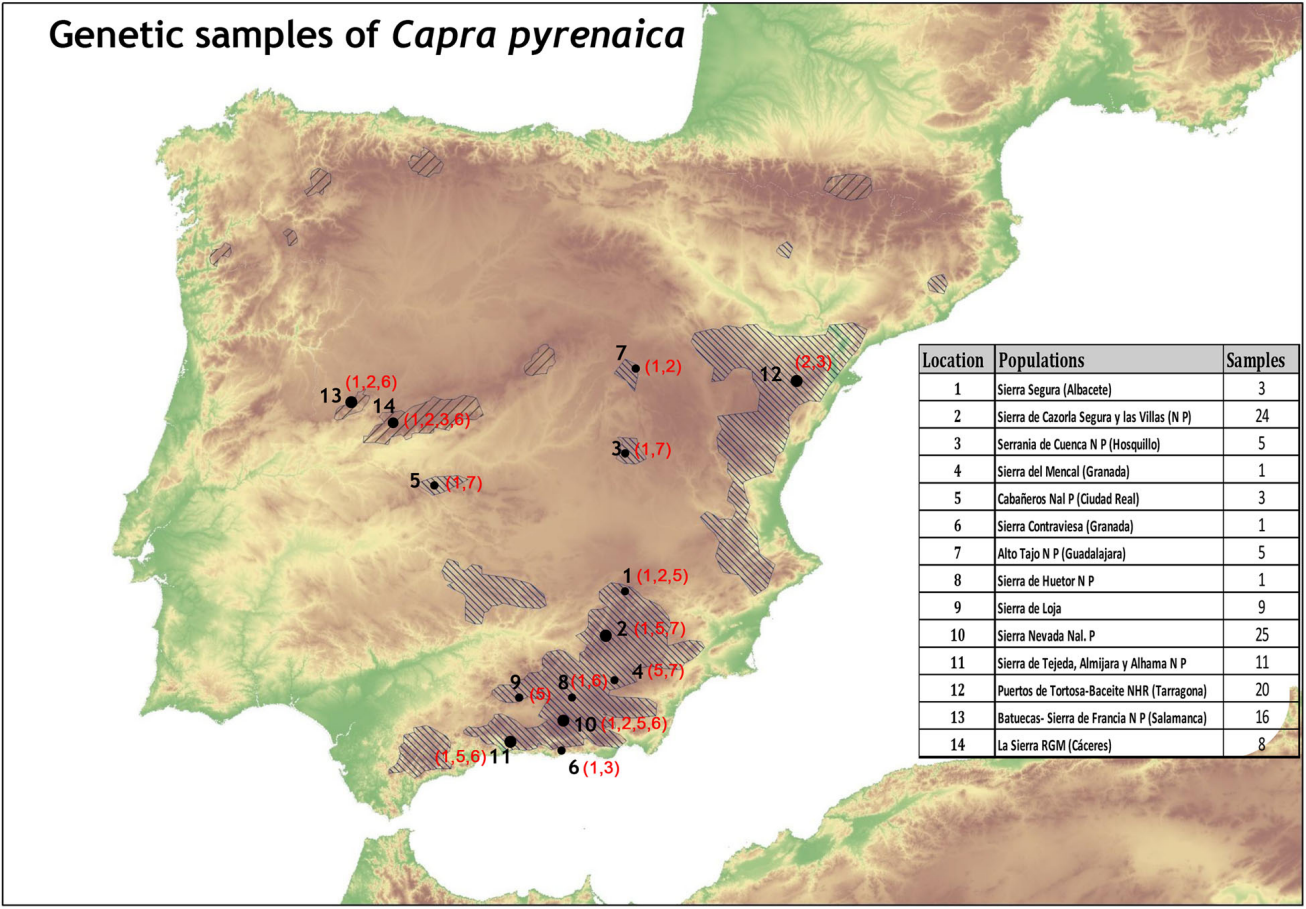
Map of the Iberian Peninsula showing the current Iberian ibex (*Capra pyrenaica*) distribution and the location of the studied populations. The MHC DRB1alleles are shown in red (DRB1*1 = 1, DRB1*2 = 2, DRB1*3 = 3, DRB1*5 = 5, DRB1*6 = 6, and DRB1*7 = 7). The populations in the Sierra Nevada National Park (10), Puertos de Tortosa and Beceite National Hunting Reserve (12), and Batuecas-Sierra de Francia Natural Park (13) have previously been studied by Alasaad et al. [23]

We collected 132 Iberian ibex samples from several Spanish populations of the surviving recognized subspecies, *C. p. hispanica* and *C. p. victoria*, in 2014–2016 (Tables 1 and 2, and Fig. 1). These samples consisted of tissue obtained from legally hunted, naturally deceased or anesthetized animals. Tissue samples were stored in 70% ethanol at − 20 °C before genomic DNA extraction with a commercial kit (NucleoSpin® Tissue; QIAGEN) following the manufacturer’s protocol.

**Table 1:** Demographic data of the studied Iberian ibex populations

Subespecies	Geographical location	Ever extinct?	Minimum population size (YEAR)	Current population Size (YEAR)	Number of founders (Year)	Origin of founders	Year of reintroduction/RETURN
*C.* *p* *.* *hispanica*	Sierra de Segura (Albacete)	YES	5 (1905)	800	NA	Natural expansion Cazorla	?
	Sierras de Cazorla, Segura and las Villas Natural Park (Jaén)	NEVER	5 (1905)	1800–2000	NA	NA	NA
	Serranía de Cuenca Natural Park, El Hosquillo	YES	10 (1964)	> 500	10	Sierras de Cazorla, Segura and las Villas Natural Park (Jaén)	1964
	Sierra del Mencal	YES	5 (1905)	150–200	NA	Natural expansion Cazorla	?
	Cabañeros National Park (Ciudad Real)	YES	15–20 (< 1995)	90	15–20	Cazorla, Gredos	< 1995
	Sierra de la Contraviesa (Granada)	YES	?	1500	NA	Natural expansion Sierra Nevada	?
	Alto Tajo Natural Park (Guadalajara)	YES	5–6 (1990)	130	5–6	Gredos	1990
	Sierra de Huétor Natural Park (Granada)	YES	?	1200	NA	Natural expansion Sierra Nevada	?
	Sierra de Loja (Granada)	NEVER	300 (1985)	1000	?	?	?
	Sierra Nevada National Park (Granada and Almería)	NEVER	450 (1960)	15,000	?	NA	?
	Sierras de Tejeda, Almijara y Alhama Natural Park (Granada and Málaga)	NEVER	750 (1962)	3000	?	?	?
	Sierras de Tortosa and Beceite National Hunting Reserve (Teruel, Castellón y Tarragona)	NEVER	450 (1966)	20,000	?	?	?
*C.* *p victoriae*	Batuecas-Sierra de Francia Natural Park (Salamanca)	YES	? (1974)	1750	34	Gredos	1974
	Sierra de Gredos- La Sierra Regional Game Reserva (Cáceres)	NEVER	? ?	10,000–13,000	?	Natural expansión Gredos	?

**Table 2:** DRB1 gene and associated OLADRB1 microsatellite alleles of the Iberian ibex samples obtained from each geographical location in Spain

Sub-species	Geographical location	Sample size	MHC DRB1 locus	OLADRB1	MHC DRB1 and OLADRB frequency (from the total) %
*C. p. hispanica*	Sierra de Segura (Albacete)	3	DRB1*1	169	66.67
			DRB1*2	159	16.67
			DRB1*5	172	16.67
	Sierras de Cazorla, Segura and las Villas Natural Park (Jaén)	24	DRB1*1	169	64.58
			DRB1*5	172	4.17
			DRB1*7	189	31.25
	Serranía de Cuenca Natural Park, El Hosquillo (originally from Cazorla)	5	DRB1*1	169	80
			DRB1*7	189	20
	Sierra del Mencal (Granada)	1	DRB1*5	172	50
			DRB1*7	189	50
	Cabañeros National Park (Ciudad Real) (originally from different populations including Cazorla)	3	DRB1*1	169	83.33
			DRB1*7	189	16.67
	Sierra de la Contraviesa (Granada)	1	DRB1*1	169	50
			DRB1*3	187	50
	Alto Tajo Natural Park (Guadalajara)	5	DRB1*1	169	50
			DRB1*2	159	50
	Sierra de Huétor Natural Park (Granada)	1	DRB1*1	169	50
			DRB1*6	185	50
	Sierra de Loja (Granada)	9	DRB1*5	172	100
	Sierra Nevada National Park (Granada and Almería)	25	DRB1*1	169	24
			DRB1*2	159	36
			DRB1*5	172	26
			DRB1*6	185	14
	Sierras de Tejeda, Almijara y Alhama Natural Park (Granada and Málaga)	11	DRB1*1	169	40.9
			DRB1*5	172	45.45
			DRB1*6	185	13.64
	Puertos de Tortosa and Beceite National Hunting Reserve (Tarragona)	20	DRB1*2	159	42.5
			DRB1*3	187	57.5
*C. p. victoriae*	Batuecas-Sierra de Francia Natural Park (Salamanca) (originally from Gredos)	16	DRB1*1	169	31.25
			DRB1*2	159	53.13
			DRB1*6	185	15.62
	“La Sierra” Regional Game Reserve (Cáceres)	8	DRB1*1	169	43.57
			DRB1*2	159	43.57
			DRB1*3	187	6.25
			DRB1*6	185	6.25

### PCR amplification and sequencing of the MHC DRB1 gene

The second exon of the DRB1 gene was sequenced using a semi-nested PCR as previously reported [28]. The PCR reaction mixture for PCR I (pre-amplification) consisted of 2 μL (25–50 ng/μl) of gDNA, 0.25 μM of each primer (using primer pairs DRB1.1 and GIo, [29]), 0.217 μM dNTP’s, 1× buffer (QIAGEN), and 0.1 μL Taq Polymerase (5 U/μL) (Hot-Start Taq DNA polymerase, QIAGEN) in a final volume of 10 μL. The samples were subjected to the following thermal profile for amplification in a 2720 Thermal Cycler (Applied Biosystems, Foster City, California): 15 min at 95 °C (initial denaturing), followed by 10 cycles of three steps of 1 min at 94 °C (denaturation), 1 min at 60 °C (annealing) and 90 s at 72 °C (extension), before a final elongation of 5 min at 72 °C. PCR blanks (reagents only) were included. We used 2 μL of the PCR-product of PCR I as a template for PCR II (semi-nested with primers DRB1.1 and DRB1.2) [29]. We employed the same PCR reaction mixture and thermal profile as in PCR I but with an annealing temperature of 65 °C and 25 (instead of 10) cycles. PCR blanks (reagents only) were included as before.

Templates of the PCR II were analyzed by Macrogene Europe Laboratories (EZ-Seq service) for sequencing. DNA sequences were aligned and edited using the software BioEdit v.7.0.9 [30]. Allele inference from heterozygous sequences was carried out with the program PHASE [31].

### OLADRB1 microsatellite genotyping

In an analysis of a microsatellite locus (OLADRB1) linked to the MHC DRB1 gene of Iberian ibex, Alasaad et al. [23] detected strong linkage disequilibrium between these loci. The allele length polymorphism at OLADRB1 was unambiguously linked to a particular DRB1 allele. This allowed the development of a DRB-STR matching method for the simple and relatively inexpensive protocol for MHC DRB1 genotyping. In our present study we used the same methodology to identify the OLADRB1 microsatellite linked to the newly discovered MHC DRB1 haplotype.

The PCR experiments were conducted using 3 μL gDNA, 0.1 μM of each OLADRB1 primers [29, 32], 0.2 μM dNTP’s, 1× buffer (QIAGEN), and 0.15 μL Taq Polymerase (5 U/μL) (Hot-Start Taq DNA polymerase, QIAGEN) in a final volume of 15 μL. PCR was performed with fluorescence-conjugated forward primer using 6-carboxyfluorescein (6-FAM). After an initial denaturation step of 15 min at 95 °C, the samples were processed through 35 cycles consisting of 30 s at 94 °C, 45 s at 60 °C and 90 s at 72 °C, followed by a terminal elongation step of 7 min at 72 °C.

Using 96-well plates, aliquots of 12 μL of formamide with LIZ size standard (5 μl LIZ-500 and 500 μl Hi-Di formamide, Applied Biosystems, Foster City, California) and 1 μL PCR product were analyzed on an ABI Prism 310 Genetic Analyzer (Applied Biosystems, Foster City, California). Allele sizes and genotypes were determined using GeneMapper 3.7 (Applied Biosystems) followed by manual proofreading.

### Molecular analyses

Genbank blast searches matching up to a 96% identity were downloaded and included in the phylogenetic analyses. For comparative purposes all the sequences used in the Amills et al. [22] study were included. Sequences were aligned in Seaview v.4.2.11 [33] with ClustalW2 [34] default settings. The best substitution model for the Bayesian inference (BI) analysis was identified using the Bayesian information criterion (BIC) in jModeltest v.2 [35]. MrBayes v.3.2.6 [36] was run with default priors and Markov chain settings, as well as with random starting trees. Runs consisted of four chains of 20,000,000 generations that were sampled every 10,000 generations. After a number of generations, a plateau with 10% of the trees derived from the analyses discarded during the burn in was reached. A maximum likelihood (ML) approach executed with the software RAxML v7.0.4 [37] with the default settings was used to estimate the phylogenetic relationships among haplotypes for each locus. The best-fitting model for the phylogenetic analyses was the HKY + G (−lnL = 1792.68621, BIC = 4112.534648). All the analyses were performed through the CIPRES platform [38], Additional file 1.

### Graphical image

The map used in Fig. 1 was prepared using political boundaries and USGS data distributed by the Land Processes Distributed Active Archive Center (LP DAAC), located at USGS/EROS, Sioux Falls, SD. http://lpdaac.usgs.gov [39]. Copyright permissions for these sources are not required.

## Results and discussion

We increased the sampling size to include previously unstudied Iberian ibex populations and discovered a new allele of the MHC DRB1 locus in four isolated populations in southern Spain, namely in Sierras de Cazorla, Segura and las Villas Natural Park (SCSVNP), El Hosquillo in Serranía de Cuenca Natural Park, Sierra del Mencal, and Cabañeros National Park (Table 2 & Fig. 1). The new allele was denominated MHC DRB1*7 (Genbank accession KY597633). This finding demonstrates greater genetic diversity in this species than previously thought (only five persistent alleles, [22, 23]), which supports our hypothesis that small and isolated wild animal populations hide unexpected genetic diversity.

**Fig. 2:**
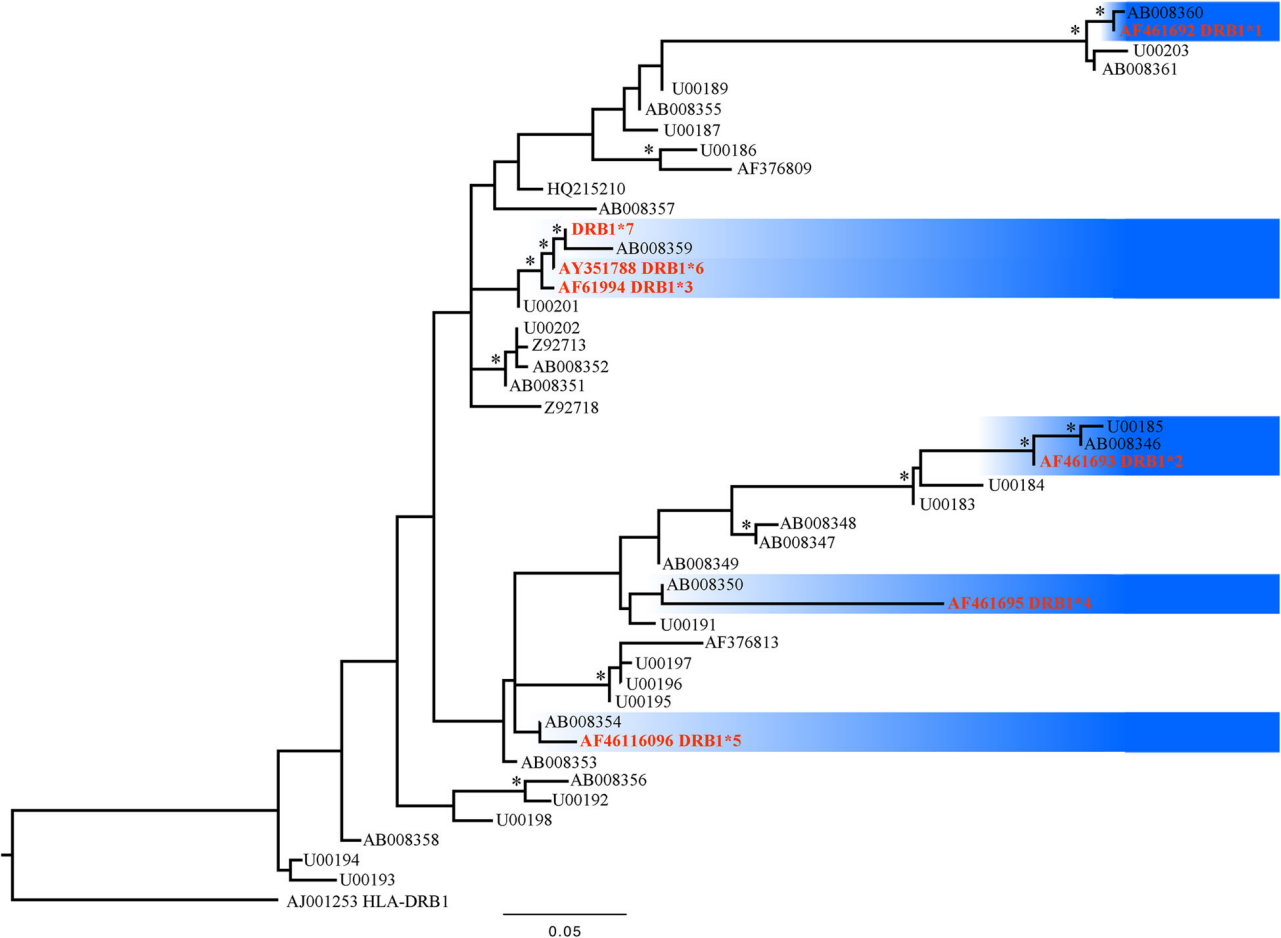
Best maximum likelihood (ML) tree for the MHC (257 bp) gene fragment. *Capra pyrenaica* haplotypes are marked in red and their clades in blue. An asterisk (*) on nodes denotes posterior probabilities (Pp) recovered from the Bayesian analysis and bootstrap support from the ML bipartition tree (≥ 95%), respectively

The aminoacid reading frame was the same for *Capra pyrenaica* DRB1.3 (AF461694), DRB1.6 (AY351788) and AB008359 (*C. hircus*). As expected given the similarity of the data, the phylogenetic analyses recovered a tree topology and paraphyly of the DRB1 haplotypes similar to the findings of Amills et al. [22]. The new haplotype Capy-DRB1*7 is grouped with *C. hircus* and *C. pyrenaica* DRB1*3 and BRB1*6. The node support was weaker in the ML than in the Bayesian analyses but the Bayesian posterior probability for this former clade was supported above 0.95. Together, this suggests good confidence in the grouping of DRB1*7 (Fig. 2).

The ibex populations in El Hosquillo and Cabañeros National Park were originally founded with a limited number of ibexes translocated from SCSVNP; Sierra del Mencal is a small mountain range 25 km from the south-western border of SCSVNP but within the dispersal range of the species. All in all, this distribution suggests that the new discovered allele originated from SCSVNP. In the late 1980s, the Iberian ibex in SCSVNP suffered a catastrophic scabies outbreak and only a few hundred individuals survived from the pre-epidemic herd of over 12,000 individuals [40].

The new discovered allele is not expected to contribute to greater fitness in *C. p. pyrenaica* since it codes for the same protein as the existing MHC DRB1*6. Further sequences of the whole gene are still needed to make a full comparison between these two alleles (MHC DRB1*6 and MHC DRB1*7). The new allele, MHC DRB1*7, is identical to one reported in the domestic Saanen goat (*C. aegagrus hircus*) (Genbank accession number U00200; [41]). Trans-species alleles for the MHC DRB1 gene have already been reported in closely related mountain ungulates such as the southern and northern chamois (*Rupicapra pyrenaica* and *R. rupicapra,* respectively) [42]. Two hypotheses could explain this similarity: I: the result of ancient polymorphism maintained by balancing selection, or II: introgression from domestic goats through hybridization [26]. Our data seem to support the introgression hypothesis since the newly discovered allele was only found in a single isolated population (and a few herds derived from it), and because hybridization between Iberian ibex and domestic goat has already been reported in the region [24]. Introgression and hybridization reports are not uncommon in Caprine species. Recent work on alpine ibex DRB genes found them to be homozygous for the goat-type DRB exon 2 alleles and almost identical to domestic goats (*Capra aegagrus hircus*). The authors of this study [43] conclude that the MHC is susceptible to adaptive introgression between species through balancing selection [44] and that introgression may well be an underappreciated mechanism generating extraordinary genetic diversity at the MHC [45]. In a few cases, hybrids between *Capra ibex ibex* and domestic goats have been reported in captivity [46] and genetically proved in the wild [47]. Hybridization between Iberian ibex (*Capra pyrenaica*) and domestic goats in the wild has also been reported [24].

Ovine and caprine populations have a great socio-economic importance in this area; censuses in SCSVNP have estimated that there are 85,100 sheep and 13,200 goats within its boundaries, of which with c. 60% is devoted to pasture (Data from Consejería de Agricultura, Pesca y Desarrollo Rural). Today, the traditional seasonal migration of cattle by farmers is now in decline but caprine production is on the increase, mainly in the southern sector of the park. These circumstances favour contact between Iberian ibex and domestic goats.

Nevertheless, Quemere et al. [8] suggest that genetic drift is the main contemporary evolutionary force shaping immunogenetic variation within populations. These authors, in contrast to the classical view, found that some genes involved in microparasite recognition continue to evolve dynamically in roe deer (*Capreolus capreolus*) in response to pathogen-mediated positive selection. In fact, high recombination rates are suspected to occur in a number of ungulate species [7]. On the other hand, low MHC variation does not seem to be the cause of disease susceptibility and demographic decline in bighorn sheep (*Ovis canadensis*) and, moreover, this variation is thought to be functionally significant and maintained by balancing selection [48].

The MHC DRB1*1 was the most frequent (35.23%) allele in the studied populations, followed by MHC DRB1*2 (24.62%), MHC DRB1*5 (17.05%), MHC DRB1*3 (9.47%), MHC DRB1*7 (7.2%) and MHC DRB1*6 (6.44%). MHC DRB1 alleles were distributed randomly without any clear longitudinal or latitudinal patterns. MHC DRB class II diversity in wild ungulates decreases with increasing latitude, possibly as a result of lower parasite diversity at higher latitudes [9]. However, this does not seem to be the case of the Iberian ibex, most likley due to the relatively small distribution area of this species.

Analysis of microsatellite locus (OLADRB1) linked to the new MHC DRB1*7 gene detected absolute linkage disequilibrium between these loci. The allele OLADRB1 with 187 bp length was unambiguously linked to the MHC DRB1*7 allele. This allowed us to develop a DRB-STR matching method for the newly discovered MHC allele.

## Conclusions

In this paper we report hidden genetic diversity in light of our discovery of a new MHC DRB1 allele in the genetically poor Iberian ibex. This newly identified allele is putatively the result of introgression from domestic goats and can be identified through a simple, newly developed protocol based on OLADRB1 microsatellite analysis. This new discovery is critical for the conservation biology of the Iberian ibex since it directly affects the identification of the units of species that should be managed and conserved separately (Evolutionarily Significant Units: ESU).

## Additional file

Additional file 1: Nexus file of the phylogenetic alignment. (ZIP 1 kb)

## Abbreviations

bp: Base pair
ESUs: Evolutionarily Significant Units
MHC: Major Histocompatibility Complex
STR: Short Tandem Repeat
